# Frequency of Retinopathy of Prematurity in Premature Neonates with a Birth Weight below 1500 Grams and a Gestational Age Less than 32 Weeks: A Study from a Tertiary Care Hospital in a Lower-Middle Income Country…

**DOI:** 10.1371/journal.pone.0100785

**Published:** 2014-07-02

**Authors:** Arjumand Sohaila, Shiyam Sunder Tikmani, Iqtidar Ahmed Khan, Huba Atiq, Ali Syed Muhammad Akhtar, Prem Kumar, Kishwer Kumar

**Affiliations:** 1 Department of Paediatrics and Child Health, Aga Khan University Hospital, Karachi, Pakistan; 2 Department of Accident and Emergency, Aga Khan University Hospital, Karachi, Pakistan; University of Utah (Salt Lake City), United States of America

## Abstract

**Introduction:**

Retinopathy of prematurity (ROP) is a treatable cause of blindness in neonates. In Pakistan, ROP is often not recognized early because screening and treatment programs are not yet in place in most neonatal units, even in tertiary care hospitals. It is hoped that this report will help inform medical professionals of the magnitude of the problem and help to design appropriate management strategies.

**Objectives:**

The aim was to determine the frequency of ROP in premature and very low birth weight (BW) neonates (BW<1500 g and gestational age (GA) <32 weeks).

**Study Design:**

Cross-sectional study.

**Study Setting:**

Neonatal intensive care unit (NICU) of a tertiary care hospital in Karachi, Pakistan.

**Study Duration:**

From June 2009 to May 2010.

**Subjects and Methods:**

Neonates with a Birth weight (BW) <1500 g and Gestational Age (GA) <32 weeks who were admitted to the NICU and received an eye examination, or were referred for a ROP eye examination as an outpatient, were included in the study. GA was estimated from intrauterine ultrasound findings. Neonates with major congenital malformations, syndromes or congenital cataracts or tumors of the eyes, and those that died before the eye examination or did not attend the out patients department for an eye examination, were excluded. The neonatal eye examination was performed by a trained ophthalmologist at 4 or 6 weeks of age.

**Results:**

Out of 86 neonates, ROP was identified in nine neonates (10.5%) at the first eye examination. ROP was significantly associated with BW (P = 0.037), GA (P = 0.033), and chronological age (P<0.001).

**Conclusions:**

we identified ROP in 10.5% of neonates at first eye examination. Significant associations between ROP and a GA<32 weeks and a BW<1500 g were also observed.we also stress that serial follow-up of neonates at risk for ROP is important when making a final diagnosis.

## Introduction

According to World Health Organization (WHO) estimates, there are 1.4 million [1] blind children worldwide, two-thirds of whom live in developing countries [1]. Retinopathy of prematurity (ROP) is the cause of blindness in about 50,000 of these children [2]. ROP is a treatable, vascular proliferative disorder that affects the incompletely vascularized retina in premature neonates [3]. Neonates with ROP are prone to develop visual complications, both structural and functional in long terms. Structural complications include refractive errors and strabismus whereas functional complications include visual dysfunction from mild to severe, even complete blindness, reduced contrast sensitivity, visual field defects and abnormal color vision and perception [4]. Excellent neonatal care can prevent most cases of ROP and blindness, but neonates who develop even minimal disease need to be identified early to prevent loss of vision [5]. ROP is a condition of the developing retinal vascular system; the incidence and severity of ROP are highly correlated with the degree of prematurity at birth [3], [6], [7], [8]. Nearly all cases occur in neonates with a BW<1500 grams and GA<32 weeks [3], [6], [8].

The incidence of ROP and blindness is much higher in developing countries than in developed countries [1], [2], [8]. The reason for this include 1) high rates of premature birth, 2) lack of ROP awareness, lack of skilled personnel, or financial constraints, and 3) lack of screening and treatment programs in most neonatal units [8], [9]. Pakistan is a developing country with poor health resources; thus, because screening and treatment programs are not yet in place, ROP is often not recognized. The true incidence of ROP in Pakistan is not known because no large, multicenter prospective studies have been conducted. In light of the background information and given the importance of ROP as a much neglected disease in our society, we conducted a prospective study at the Aga Khan University Hospital (AKUH) to systematically collect and report data on the frequency of ROP in premature neonates. The aim was to increase awareness among medical professionals and to facilitate the design of appropriate management strategies. We identified ROP in 10.5% of neonates at the first eye examination. A previous study at our institution performed serial eye examinations in neonates at risk for ROP at 2-week intervals until the retina was fully vascularized and reported an incidence of 32.4% [10]. Therefore, we stress the importance of early, serial eye examinations as a part of a strategy to control ROP.

## Materials and Methods

### Patients and study design

This article is based on a dissertation, and the study protocol was approved by the Research Evaluation Unit of College of Physicians and Surgeons of Pakistan. Neonates fulfilling the inclusion criteria were enrolled in the study after their parents or guardians provided written informed consent.

This was a prospective study carried out in the neonatal intensive care unit (NICU) at AKUH from June 2009 to May 2010. Neonates with a BW<1500 g and a GA<32 weeks who were admitted to the NICU during the study period and given an eye examination, or clinic patients referred for ROP and given an eye examination, were eligible for inclusion. The inclusion criteria were derived from established screening criteria at AKUH, which were adapted from recommendations by the British Royal College of Ophthalmologists [11]. For example, rather than screening at a GA of <31 weeks, we screened at <32 weeks as per hospital protocol. Gestational age was assessed on the basis of intrauterine ultrasound findings. Neonates who met the inclusion criteria but had major congenital malformations, syndromes or congenital cataracts or tumors of the eyes, and those who died before the eye examination could be conducted or were not referred for an eye examination, were excluded. Eye examinations were performed by a trained ophthalmologist. Before the examination, eye drops containing 0.5% tropicamide and 0.5% phenylephrine were instilled three times (1 minute apart) to dilate the pupils. Indirect ophthalmoscopy was performed using a binocular indirect ophthalmoscope. A lid speculum and sclera depressors were used routinely. All examination results were recorded using a predesigned form.

### Statistical analysis

A nonprobability purposive sampling technique was applied to the study sample. Approximately 120 premature neonates are admitted to the NICU at AKUH every year. Assuming a 23.5% prevalence (P) of ROP among the study population (as defined by the inclusion criteria), with an acceptable error of 9% and a 95% confidence level, we used the following equation to calculate the required sample size(*n*)(*n* = *Z* ^2^_1−α/*S*_ *P*(1−*P*)/*d* ^2^) From this equation, n = 86. The expected prevalence was assumed to be similar to that in India, a neighboring country with socioeconomic and demographic characteristics comparable with those in Pakistan. The reported prevalence rates of ROP in India range from 20% to 27% (mean, 23.5%) [12].

Data entry and analysis was done using SPSS version 16.0 (IBM SPSS Statistics, USA). The results are expressed as the mean and standard deviation for continuous variables (e.g., weight and GA) and as numbers and percentages for categorical variables (e.g., gender and presence or absence of ROP). Data analysis was based on patient groups i.e., Groups 1, 2 and 3 as described above. The Chi-squared test was used to compare categorical variables. Fisher's exact test was used when the expected cell count was <5. Student “t” test was applied to compare continuous variables. P values<0.05 was considered significant.

## Results

A total of 86 neonates were enrolled in the study. Seven (8.1%) were from the NICU and 79 (91.9%) were from the clinic. Of these, 46 (53.5%) were male and 40 (46.5%) were female (1.15∶1). Forty-six neonates (53.5%) had a GA of <32 weeks and 40 (46.5%) had a GA>32 weeks but BW<1500 g. Forty-five neonates (52.3%) had a BW<1500 g and 41 (47.7%) had a BW>1500 g but GA<32 weeks. Mean birth weight for GA group >32 weeks was 1295 g whereas in group <32 weeks was 1989 g with overall mean of 1.64±0.98. Fifty-eight neonates (67.4%) were 4 weeks of age at the time of examination. Nine of the neonates (10.5%) were diagnosed with ROP. Among them one patient required immediate treatment as with Type 1 ROP (high risk). The characteristics of the neonates enrolled in the study are shown in Tables 1 and 2.

**Table 1:** Characteristics of the neonates enrolled in the study.

Characteristics		Number (N = 86)	Percentage (%)
**Chronological age**	4 weeks	58	67.4
	5 weeks	21	24.4
	6 weeks	7	8.1
**Gender**	Male	46	53.5
	Female	40	46.5
**Gestational age (Mean)**	33.57±1.2 weeks		
	Less than 32 weeks	46	53.5
	More than 32 weeks	40	46.5
**Birth weight (Mean)**	1.84±0.98 kg		
	Less than 1.5 kg	45	52.3
	More than 1.5 kg	41	47.7
**Place of eye examination**	Clinic	79	91.9
	NICU	7	8.1

NICU = Neonatal intensive care unit; kg = kilograms.

**Table 2:** Characteristics of the neonates with ROP.

Retinopathy of prematurity (ROP)	Number (N = 86)	Percentage (%)
**Present**	9	10.5
**Absent**	77	89.5
**Group 1: No ROP**	77	89.5
**Group 2: ROP not requiring treatment**	8	9.3
**Group 3: ROP requiring treatment**	1	1.2

NICU = Neonatal intensive care unit; ROP = Retinopathy of Prematurity.

There were no significant associations of the presence of ROP with gender (P = 1.000) or site of admission (NICU or clinic) (P = 0.552). However, there were significant associations of the presence of ROP and GA (P = 0.033), BW (P = 0.037), and chronological age (P<0.001). Table 3 shows the comparison between ROP and the subjects' clinical characteristics.

**Table 3:** Association between ROP and patient (N = 86) and study variables.

Characteristics		Retinopathy of Prematurity		P -value*
		Present	Absent	
**Chronological age**	4 weeks	2	56	<0.001
	5 weeks	3	18	
	6 weeks	4	3	
**Gender**	Male	5	41	1.000
	Female	4	36	
**Gestational age**	Mean	32.86±1.3 weeks	35.71±2.3 weeks	0.047
	Less than 32 weeks	8	38	0.033
	More than 32 weeks	1	39	
**Birth weight**	Mean	1.54±0.38 kg	1.94±1.3 kg	0.037
	Less than 1.5 kg	8	37	0.031
	More than 1.5 kg	1	40	
**Place of eye examination**	Clinic	8	71	0.552
	NICU	1	6	

NICU, neonatal intensive care unit; ROP, retinopathy of prematurity; kg = kilograms.

*Fisher's exact test.

## Discussion

Advances in neonatology have led to increased survival for extremely premature neonates, ultimately increasing the number of neonates with ROP. Interestingly, the BW and GA ranges in which neonates develop severe ROP are far wider in low- and middle-income countries than in more developed and advanced countries [8]. Available worldwide data suggest that blindness due to ROP varies significantly from country to country [9]. The incidence of blindness due to ROP in the various World Bank regions was estimated by plotting blindness data against infant mortality rates (IMRs) for 1999 [9]. The plot suggests that countries fall into three distinct groups. Of these, ROP is emerging as an important cause of blindness in middle-income countries, with IMRs ranging from 9–60/1000 live births [9]. Causes include increased rates of teenage pregnancy and premature birth, intensive neonatal care units with variable standards of care, and lack of effective programs for the screening and treatment of ROP [8], [9].

We diagnosed ROP in 10.5% of cases at the time of the first eye examination. Another study conducted at AKUH performed serial eye examinations at 2 week intervals until the retina was fully vascularized, and found that the incidence of ROP was 32.4% [10]. We conclude that serial monitoring (eye examinations) of neonates at risk for ROP is important for making a final diagnosis. Although the present study was conducted on a small sample and made a limited number of observations, the results show a significant association between ROP and a BW<1500 g and a GA<32 weeks. In our study we also observed a large proportion of growth restricted babies (IUGR or SGA) as our hospital is one of the biggest tertiary care hospital of the city and a referral hospital. IUGR are associated with increased incidence of ROP compared with appropriate for gestational age [13], [14], [15].

## Conclusion

ROP is becoming a major cause of potentially preventable blindness among children in lower-middle income countries. We identified ROP in 10.5% of neonates at the time of their first eye examination and found a significant association between ROP and a GA<32 weeks and a BW<1500 g. Therefore, we stress the importance of follow-up eye examinations in neonates at risk for ROP, particularly as a previous follow-up study that conducted eye examinations at 2 week intervals until the retina was fully vascularized found that the incidence increased to 32.4% [10].

### Copyright Disclosure Statement

We, the undersigned coauthors of this article, have contributed significantly to, and share in the responsibility for this article. The undersigned stipulate that the material submitted to PLoS One is new, original, and has not been submitted to another publication for concurrent consideration.

We also attest that the research carried out in human subjects and reported in this manuscript was in compliance with the regulations of our institution and with generally accepted guidelines governing such work.

We further attest that we have herein disclosed any and all financial or other relationships that could be construed as a conflict of interest and that all sources of financial support for this study have been disclosed and are indicated in the acknowledgement.

This manuscript has been read and approved by all authors.
